# Investigation of the Residual Stress in a Multi-Pass T-Welded Joint Using Low Transformation Temperature Welding Wire

**DOI:** 10.3390/ma14020325

**Published:** 2021-01-10

**Authors:** Zhongyuan Feng, Ninshu Ma, Seiichiro Tsutsumi, Fenggui Lu

**Affiliations:** 1 Joining and Welding Research Institute, Osaka University, Osaka 567-0047, Japan; tsutsumi@jwri.osaka-u.ac.jp; 2 Graduate School of Engineering, Osaka University, Osaka 565-0871, Japan; 3 School of Materials Science and Engineering, Shanghai Jiao Tong University, Shanghai 200240, China; Lfg119@sjtu.edu.cn

**Keywords:** low transformation temperature weld metal, residual stress, martensitic transformation, reheat temperature, multi-pass welding

## Abstract

We investigated whether low transformation temperature (LTT) welding materials are beneficial to the generation of compressive residual stress around a weld zone, thus enhancing the fatigue performance of the welded joint. An experimental and numerical study were conducted in order to analyze the residual stress in multi-pass T-welded joints using LTT welding wire. It was found that, compared to the conventional welded joint, greater tensile residual stress was induced in the flange plate of the LTT welded joints. This was attributed to the reheat temperature of the LTT weld pass during the multi-pass welding. The formerly-formed LTT weld pass with a reheat temperature lower than the austenite finish temperature converted the compressive residual stress into tensile stress. The compressive residual stress was generated in the regions with a reheat temperature higher than the austenite finish temperature, indicating that LTT welding materials are more suitable for single-pass welding.

## 1. Introduction

It is well known that fusion welding has been applied to a large number of engineering components. However, the non-uniform temperature distribution and constraint during the welding process engender residual stress in the welded joints [1]. Generally, tensile residual stress is detrimental to the fatigue strength, corrosion resistance and crack resistance of welded joints [2,3,4]. In order to minimize these adverse effects, it is necessary to take appropriate measures, such as ultrasonic peening [5], TIG dressing [6], local heat treatment [7] and so on. Nevertheless, these post-weld treatments are costly and time consuming.

During the 1970s, Jones and Alberry found that the stress accumulation in steels under constraint conditions can be reduced by bainitic or martensitic transformation [8]. With the development of materials science, Murata et al., in the 1990s, successfully developed an iron-based alloy using chromium and nickel as the main alloying elements, producing a martensite start (Ms) temperature of approximately 180 °C [9]. Owing to the lower Ms temperature, this type of iron-based alloy is also referred to as low transformation temperature (LTT) alloy. The LTT alloys exploit the plasticity associated with displacive transformation in order to offset thermal contraction strain, thus mitigating the tensile residual stress around the weld zone [10,11]. Due to tensile residual stress mitigation, numerous studies have shown that LTT alloys can significantly enhance the fatigue performance of welded joints [12,13,14]. Additionally, the LTT alloys were combined with TIG dressing for the further improvement of the fatigue strength [15]. In addition to fatigue life improvement, the researchers also used the LTT alloys to reduce distortion [16] and to resist cold cracking [17]. In order to obtain an optimized residual stress around the weld zone, much attention was focused on the effects of the martensite start (Ms) temperature, which involved both experimental measurements and numerical simulation [18,19,20]. It was found that if the martensitic transformation occurred at an elevated temperature of more than 400 °C, the martensite finish (Mf) temperature would be located above the ambient temperature, resulting in a build-up tensile stress due to thermal contraction during the cooling process [21,22]. Regarding Ms temperatures lower than 80 °C, the martensitic transformation was not fully completed, which led to a smaller volume expansion strain and rendered the tensile residual stress mitigation insufficient [23]. Another relevant factor that needs to be considered is whether the interpass temperature or the reheat temperature is above or below the Ms temperature. Since a single-pass weld goes through only one thermal excursion, it is very easy to control the welding’s residual stress. In practice, most engineering structures are fabricated with multi-pass welds. Unlike a single-pass weld, multi-pass welds undergo several thermal excursions as subsequent weld passes are deposited. Whether tensile residual stress can be induced in a multi-pass welded joint is greatly dependent on the reheat temperature. Experimental and numerical studies on residual stress in multi-pass butt-welded joints have shown that, if the reheat temperature is higher than the Ms temperature, much of weld zone is left under compressive stress; otherwise, the thermally-induced residual stress increases again [24]. Of course, the specimen dimensions, welding parameters and number of weld passes also affect the reheat temperature. According to previous studies, the final weld bead usually has a lower compressive residual stress, and there are smaller residual stresses induced at the weld toe of butt-welded joints due to the strong reheat effect of the final weld pass [25]. Generally, fatigue cracks are initiated at these critical positions, such as the weld toe and the weld root. Therefore, LTT butt-welded joints with smaller residual stresses at the weld toe may still have a better fatigue life in comparison with a conventional welded joint. Apart from a butt-welded joint, the fillet welded joint, like the T-welded joint, is also one of the most common connections in welded fabrication. However, all of the previous studies have focused on the effect of the reheat temperature on butt-welded joints, rather than fillet welded joints. Hence, it is of great significance to conduct a study of the reheat effect on the residual stress in fillet-welded joints.

The aim of this paper is to investigate the effect of the reheat temperature on the residual stress in multi-pass T-welded joints using LTT welding wire. Accordingly, the residual stress distribution of the conventional T-welded joint was used for reference. In order to clarify the residual stress development in the course of the welding process, a finite-element analysis was performed using the in-house software JWRIAN [26,27]. Finally, the computed residual stresses were compared with those obtained from the experimental measurement.

## 2. Materials and Methods

**Figure 1:**
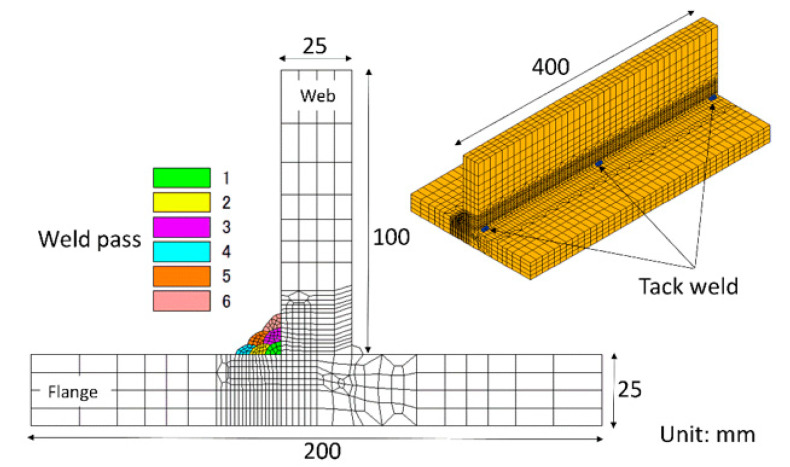
Schematic of a multi-pass T welded joint.

A schematic of a T-welded joint is displayed in Figure 1. Two T-welded joints were fabricated using a gas metal arc welding process using two types of commercial welding wires, LB-3AD and LB-47, respectively. Accordingly, the former welded joint belongs to the LTT welded joint, while the latter is the conventional welded joint. The chemical compositions of the SM490A welded plates, the filler wire and their weld metals are listed in Table 1. During the welding process, the welding current, voltage and speed were 130 A, 22 V and 120 mm/min, respectively. Meanwhile, a shielding gas, 80% Ar + 20% CO_2_, was employed throughout. Each weld pass was cooled down to an ambient temperature of 15 °C before the start of the subsequent weld pass. After the fabrication of the welded joints, residual stress measurement was conducted by means of μ-X360 FULL 2D Portable X-ray Residual Stress Analyzer (PULSTEC, Tokyo, Japan) using the cosα method [28]. In the cosα method, the signal of a diffraction ring (Debye ring) is detected by an area detector. Then, the strain *ε_α_* where *α* = 0–360° at the Debye ring is applied in order to compute the residual stress in the welded specimen, and can be expressed as follows:

(1) $$\varepsilon_{\alpha }=\frac{\sigma_{x}}{E}\left[n_{1}^{2}-\nu \left(n_{2}^{2}+n_{3}^{2}\right)\right]+\frac{\sigma_{y}}{E}\left[n_{2}^{2}-\nu \left(n_{1}^{2}+n_{3}^{2}\right)\right]+\frac{2\left(1+\nu \right)}{E}\tau_{xy}n_{1}n_{2}$$

where *n*_1_, *n*_2_ and *n*_3_ represent the orientation cosine, respectively.

(2) $$n_{1}=\cos\eta \sin\psi_{0}\cos\phi_{0}-\sin\eta \cos\psi_{0}\cos\phi_{0}\cos\alpha -\sin\eta \sin\phi_{0}\sin\alpha$$

(3) $$n_{2}=\cos\eta \sin\psi_{0}\sin\phi_{0}-\sin\eta \cos\psi_{0}\sin\phi_{0}\cos\alpha +\sin\eta \cos\phi_{0}\sin\alpha$$

(4) $$n_{3}=\cos\eta \cos\psi_{0}+\sin\eta \sin\psi_{0}\cos\alpha$$

where η, $\phi_{0}$ and $\psi_{0}$ represent the diffraction angle between the reflection line and the input X-ray, the orientation angle between the projection of the input X-ray on the sample’s surface and the axis x, and the orientation angle between the normal line of the sample and the input X-ray, respectively.

A set of four strains, $\varepsilon_{\alpha }$, $\varepsilon_{\pi +\alpha }$, $\varepsilon_{-\alpha }$ and $\varepsilon_{\pi -\alpha }$, were measured for each *α* in order to minimize the possible experimental error, which can be expressed as follows:

(5) $$a_{1}\equiv \frac{1}{2}\left[\left(\varepsilon_{\alpha }-\varepsilon_{\pi +\alpha }\right)+\left(\varepsilon_{-\alpha }-\varepsilon_{\pi -\alpha }\right)\right]$$

Then,

(6) $$a_{1}=-\frac{1+v}{E}\sigma_{x}\sin2\psi_{0}\sin2\eta \cos\alpha$$

Hence, $\sigma_{x}$ can be determined from the slope, *M*_1_, of the linear relationship between $a_{1}$ and cosα, as:

(7) $$\sigma_{x}=-\frac{E}{1+\nu }\frac{1}{\sin2\psi_{0}}\frac{1}{\sin2\eta }\left(\frac{\partial a_{1}}{\partial \cos\alpha }\right)=-\frac{E}{1+v}\frac{1}{\sin2\psi_{0}}\frac{1}{\sin2\eta }M_{1}$$

The measuring parameters of the XRD device are shown in Table 2. Owing to the geometry of the T-welded joint, it is very difficult to measure the longitudinal residual stress (parallel to the welding direction) adjacent to the weld toe. Therefore, the transverse stress (vertical with regard to the welding direction) in the flange plate was measured. In order to identify the transformation temperatures of the LTT weld metal, a dilatometric test was performed using Formaster equipment (FUJI, Tokyo, Japan). Generally, a higher heating rate leads to higher austenite start and finish temperatures, Ac1 and Ac3 temperatures [29]. Considering the repeated thermal cycles and the different reheating rates in multi-pass welds, a small heating rate of 10 °C/s was adopted for the Formaster test. In this way, the regions away from the newly-formed LTT weld pass can be taken into account due to the smaller reheating rate and lower Ac1 and Ac3 temperatures. According to the empirical equation proposed by Yurioka et al. [30], the critical cooling time *T*_M_ from 800 to 500 °C to obtain a fully martensitic microstructure is given as:

(8) $$\ln T_{M}=10.6CE-4.8$$

(9) $$CE=C+\frac{Si}{24}+\frac{Mn}{6}+\frac{Cu}{15}+\frac{Ni}{12}+\frac{Cr}{8}+\frac{Mo}{4}$$

The value of *T_M_* for a LTT weld bead is about 37 s; in other words, a cooling rate higher than 8.2 °C/s is acceptable. Hence, a solid cylindrical sample extracted from a LTT weld bead with a dimension of ϕ3.0 × 10 mm was heated from ambient temperature up to 1350 °C at a heating rate of 10 °C/s and held for 2 s, followed by continuous cooling to ambient temperature at a cooling rate of 20 °C/s.

## 3. Results and Discussion

**Figure 2:**
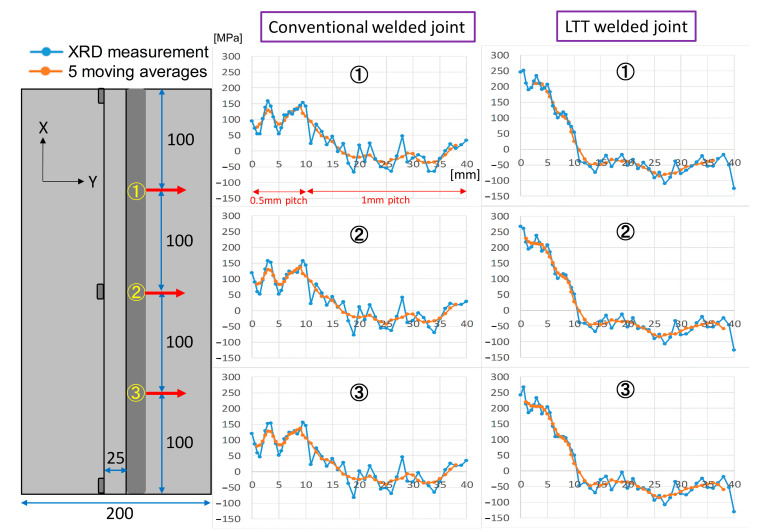
Measured residual Sy stress of the two welded joints.

In order to compare the residual stress between the conventional and LTT welded joints, as shown in Figure 2, the residual Sy stress was measured using the X-ray diffraction technique in three positions of the flange plate. The measured residual Sy stress is plotted at the right side of Figure 2, which also shows the five moving averages intended to minimize the experimental error. Evidently, the residual stress distribution is very similar among these three positions, demonstrating the high accuracy of the experimental measurement. Furthermore, the conventional welded joint has a lower tensile residual stress of about 100 MPa at the weld toe compared to that of approximately 250 MPa in the LTT welded joint. With the distance away from weld toe, the tensile residual stress in the LTT welded joint gradually decreases. Accordingly, the reheat temperature is responsible for the higher tensile residual stress induced in the LTT welded joint. In order to clarify the difference in the residual stress distribution between these two welded joints, a thermal–elastic–plastic analysis was implemented using the in-house software, JWRIAN.

**Figure 3:**
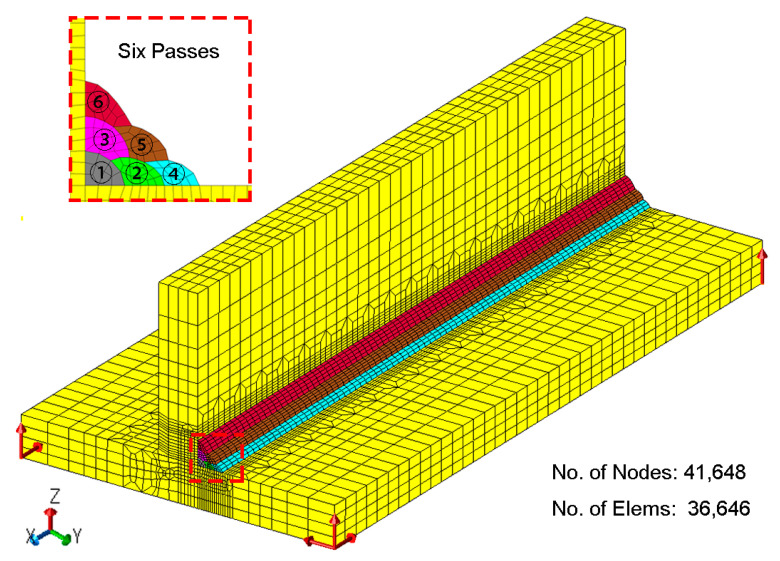
Simulation model for the computation of the residual stress.

Figure 3 shows the simulation model for the computation of the residual stress. The total numbers of nodes and elements are 41,648 and 36,646, respectively. A finer mesh of about 2 mm was adopted around the weld pass, while coarser meshes were utilized in regions away from weld zone, with a maximum size of 8 mm. The boundary condition was defined by red arrows, which denoted the fixed displacement in the corresponding direction. Six weld passes were deposited at one side of the T-welded joints, and each weld pass was cooled to an ambient temperature of 15 °C before the start of the next weld pass. The welding parameters of the welding simulation were the same as the experiment. In addition, the conventional von Mises’ elastoplastic flow theory was taken into account:

(10) $$d\varepsilon =d\varepsilon^{e}+d\varepsilon^{p}+d\varepsilon^{\mathrm{th}}+d\varepsilon^{\mathrm{tr}}$$

where $d\varepsilon^{e},d\varepsilon^{p},d\varepsilon^{\mathrm{th}}\;\mathrm{and}\;d\varepsilon^{\mathrm{tr}}$ represent the increments of elastic strain, plastic strain, thermal strain and transformation plastic strain, respectively. Furthermore, the isotropic hardening model was used for the computation of the residual stress.

**Figure 4:**
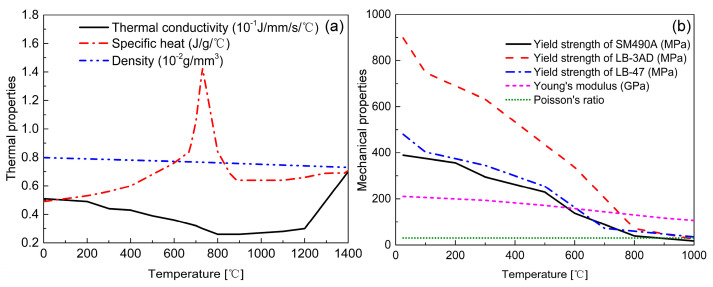
Material properties of the LTT weld metal: (**a**) thermal properties; (**b**) mechanical properties.

**Figure 5:**
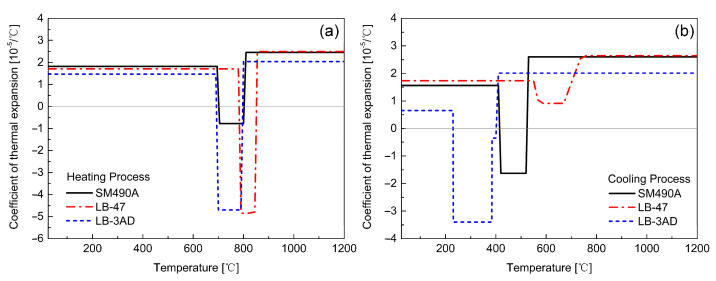
CTE of the welded components during the heating and cooling processes: (**a**) heating process; (**b**) cooling process.

Figure 4 shows the thermal and mechanical properties of the LTT weld metals. The thermal properties were obtained from other published papers as references [31,32]. Except for the mechanical properties at room temperature, the other temperature-dependent mechanical properties of LB-47 and LB-3AD were computed using thermodynamic software, JMatPro. The mechanical properties of SM490A were experimentally measured based on the JIS G3106 Standard. In addition, Figure 5 displays the transient coefficients of thermal expansion (CTE) of the welded components, as measured by the dilatometric test. The CTE is given by [33]:

(11) CTE=dε/ΔT

(12) $$d\varepsilon =\Delta L/L_{0}$$

where $L_{0}$ is the sample length at room temperature and ΔL is the temperature-dependent length change.

### 3.1. Reheat Temperature of LTT Weld Passes

**Figure 6:**
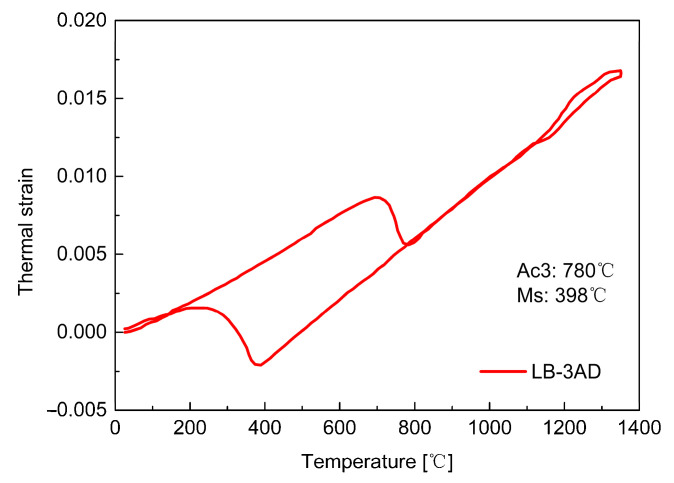
Dilatometric results of the LTT weld metal.

**Figure 7:**
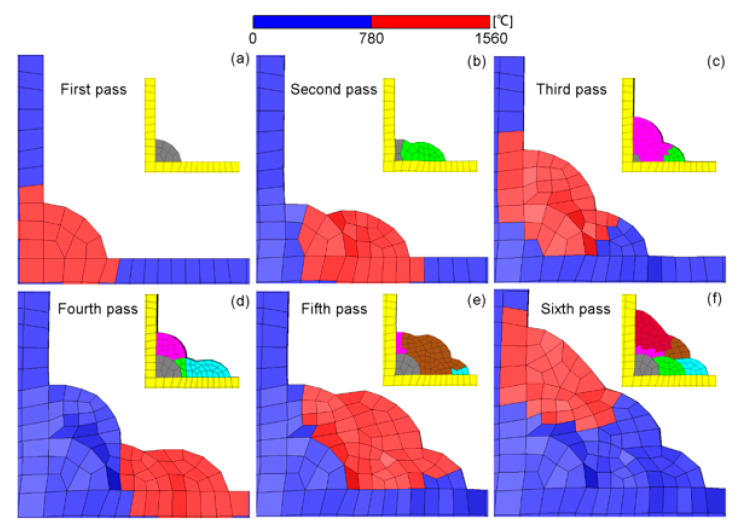
Temperature distribution during the welding process for the redefinition of the CTE of LTT bead: (**a**) first pass; (**b**) second pass; (**c**) third pass; (**d**) fourth pass; (**e**) fifth pass; (**f**) sixth pass.

In multi-pass welding, the formerly-formed weld beads undergo different thermal cycles. As mentioned before, the reheat temperature plays an indispensable role in the residual stress development in a LTT welded joint. Hence, it is critical to evaluate the reheat temperature distribution induced by these thermal cycles in the formerly-formed weld beads. Additionally, the reheat temperature has a strong relationship with the Ac3 temperature. According to the dilatometric results of the LTT weld metal shown in Figure 6, the Ac3 temperature during the heating process is about 780 °C. For regions with a reheat temperature less than 780 °C, martensite cannot fully transform into austenite, resulting in an increase in the thermally-induced tensile residual stress. In other words, the CTE of this region during the cooling process is the same as that of the heating process. If the reheat temperature exceeds 780 °C, martensite will fully transform into austenite during the heating process, and then the austenite transforms into martensite again during the cooling process, thus producing the beneficial compressive residual stress. Figure 7 shows the temperature distribution of the weld passes after each welding. According to the temperature distribution and Ac3 temperature, the regions with a temperature higher than 780 °C can be defined as the ‘real’ LTT weld zone, which can produce beneficial compressive residual stress after each welding. Conversely, the regions with temperatures less than 780 °C are defined as the ‘non-real’ LTT weld zone. Correspondingly, the CTEs of the ‘non-real’ and ‘real’ LTT weld metals are redefined again for the residual stress computation, as shown in Figure 7.

### 3.2. Comparison of the Residual Stresses between the Welded Joints

**Figure 8:**
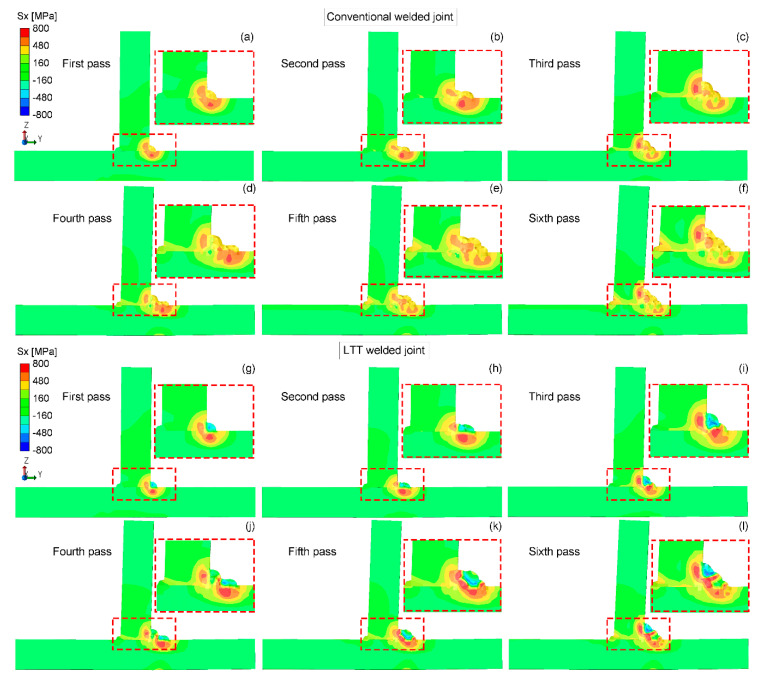
Residual Sx stress distribution in the mid-section of the conventional and LTT -welded joints: (**a**,**g**) first pass; (**b**,**h**) second pass; (**c**,**i**) third pass; (**d**,**j**) fourth pass; (**e**,**k**) fifth pass; (**f**,**l**) sixth pass.

Figure 8 shows the longitudinal residual Sx stress distribution in the mid-section of the conventional and LTT welded joints. It can be seen in the conventional welded joint that the whole weld zone is accompanied by a relatively higher tensile residual stress. For the LTT welded joint, compressive residual stress is mainly produced in the newly-formed LTT weld bead and the weld zone with a reheat temperature higher than the Ac3 temperature, balanced by the tensile stress adjacent to this region. Once the reheat temperature is up to the Ac3 temperature, the microstructure of the formerly-formed weld zone transforms into austenite again in the heating process, and then the martensitic transformation occurs during the cooling process, thus producing compressive residual stress. Owing to welding sequence, the compressive residual stress of the weld passes formed first converts into tensile stress if the reheat temperature is less than the Ac3 temperature. Therefore, greater tensile residual stress is generated at the weld toe of weld pass 4, while lower stress is found at the weld toe of weld pass 6. Furthermore, the residual stress near the tack weld in both welded joints increases with the number of weld passes, and the conventional welded joint has a larger tensile stress.

**Figure 9:**
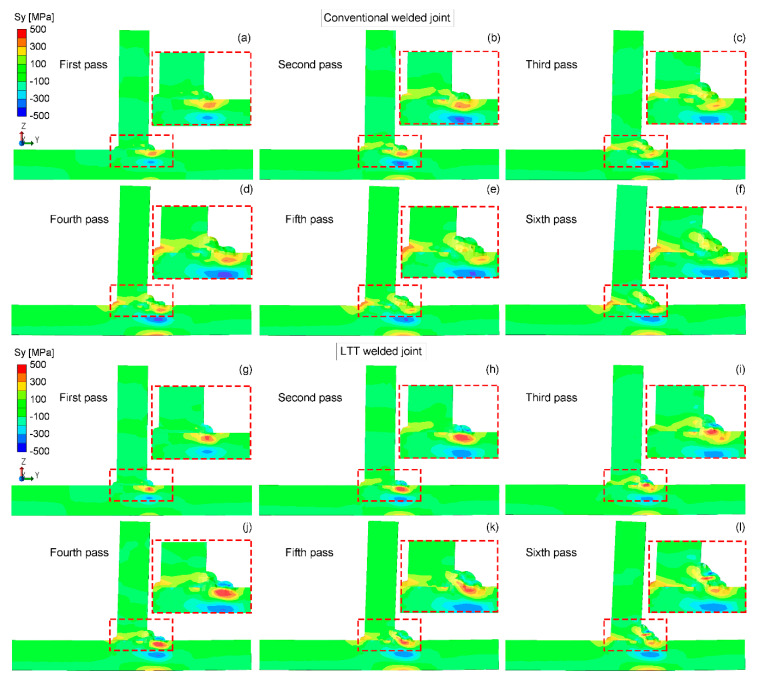
Residual Sy stress distribution in the mid-section of the conventional and LTT welded joints: (**a**,**g**) first pass; (**b**,**h**) second pass; (**c**,**i**) third pass; (**d**,**j**) fourth pass; (**e**,**k**) fifth pass; (**f**,**l**) sixth pass.

Figure 9 shows the residual Sy stress distribution in the mid-section of the conventional and LTT welded joints. Compared to the longitudinal residual Sx stress, both welded joints have lower tensile residual Sy stresses. In the conventional welded joint, most of the weld zone is left with a greater tensile stress. For the LTT welded joint, similar to the longitudinal residual Sx stress distribution, the compressive residual Sy stress is mainly produced in the newly-formed LTT weld zone with a reheat temperature higher than the Ac3 temperature, balanced by the tensile stress near this region. Due to the welding sequence, the residual stress state of those weld passes formed first converts from compressive to tensile because the reheat temperature is lower than the Ac3 temperature. Hence, greater tensile residual stress is also induced at the weld toe of weld pass 4, while compressive stress is produced at the weld toe of weld pass 6. Moreover, the residual stress near the tack weld in both welded joints builds up gradually during the welding process, and the conventional welded joint also has a greater tensile stress.

**Figure 10:**
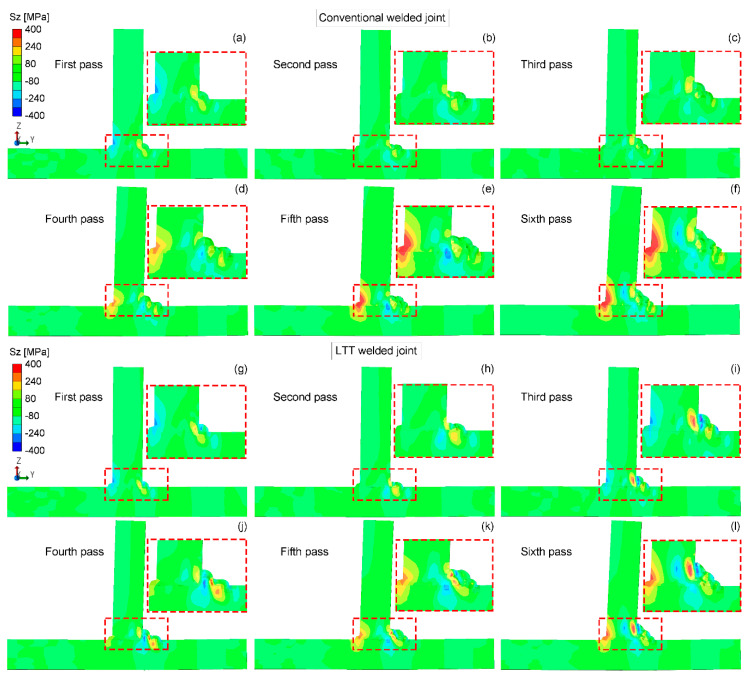
Residual Sz stress distribution in the mid-section of the conventional and LTT welded joints: (**a**,**g**) first pass; (**b**,**h**) second pass; (**c**,**i**) third pass; (**d**,**j**) fourth pass; (**e**,**k**) fifth pass; (**f**,**l**) sixth pass.

Figure 10 shows the residual Sz stress distribution in the mid-section of the conventional and LTT welded joints. Compared to the residual Sx and Sy stresses, both welded joints have lower tensile residual Sz stresses. Meanwhile, residual Sz stress in the conventional welded joint is distributed more uniformly, as it was against the LTT welded joint. Similarly, the compressive residual stress is mainly produced in the newly-formed LTT weld zone with a reheat temperature higher than the Ac3 temperature, balanced by the tensile stress close to this region. The welding sequence has the same effect on the residual stress state of the weld passes, leading to a greater tensile residual stress at the weld toe of weld pass 4 and compressive stress at the weld toe of weld pass 6. Additionally, the conventional welded joint has a greater tensile residual stress near the tack weld in comparison with the LTT welded joint. This can be attributed to the greater tensile residual stress induced in the conventional weld bead, which results in a greater distortion of the web plate.

**Figure 11:**
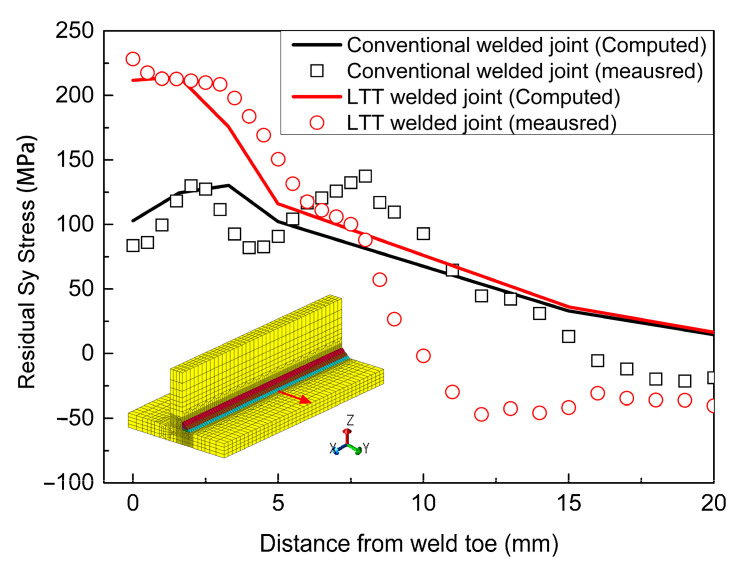
Computed residual Sy stress versus the experimental result.

**Figure 12:**
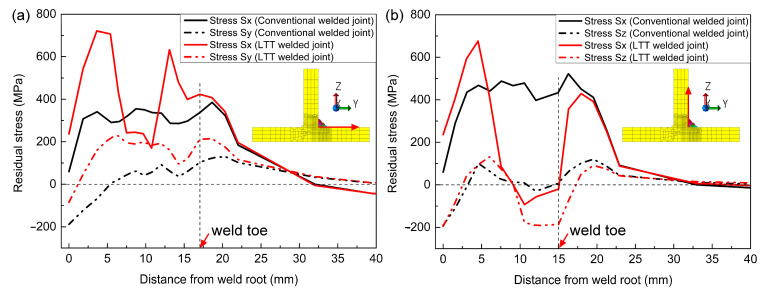
Comparison of the simulated residual stresses along the red line: (**a**) on the flange plate; (**b**) on the web plate.

Figure 11 compares the computed residual Sy stress with the measured stress in order to verify the computation results. It can be seen that the computed residual stresses are in good agreement with the experimental measurement adjacent to the weld toe. With a distance from weld toe longer than 8 mm, there exists an error between the measurement and the computation. This can be attributed to the initial stress of the welded plate induced before the welding, which is highly dependent on the manufacturing processes of the welded plate, such as bending or rolling [34,35]. In order to quantitatively compare the residual stress between these two welded joints, Figure 12 shows the simulated residual stress distribution starting from the weld root along the two red lines on the flange and web plates. Evidently, except for the residual Sz stress, the residual Sx and Sy stresses at the weld root of LTT welded joint are greater than those of the conventional welded joint. Due to the effect of the reheat temperature, as depicted in Figure 12a, the weld toe of weld pass 4 in the LTT welded joint has greater tensile residual Sx and Sy stresses than those of the conventional welded joint. Conversely, as shown in Figure 12b, the weld toe of weld pass 6 in the LTT welded joint has compressive residual Sx and Sz stresses of −186 MPa and −20 MPa, respectively. On the other hand, the residual Sx and Sz stresses at the weld toe of weld pass 6 in the conventional welded joint are 433 MPa and 6 MPa, respectively.

**Figure 13:**
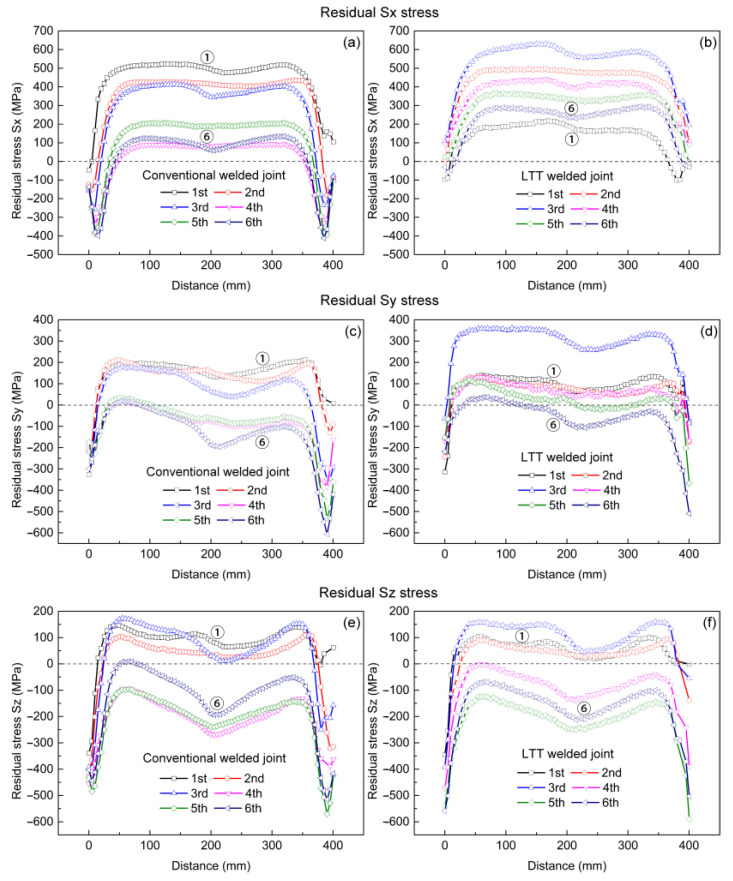
Simulated residual stress at the weld roots of the conventional and LTT welded joints after each welding: (**a**) residual Sx stress in conventional welded joint; (**b**) residual Sx stress in LTT welded joint; (**c**) residual Sy stress in conventional welded joint; (**d**) residual Sy stress in LTT welded joint; (**e**) residual Sz stress in conventional welded joint; (**f**) residual Sz stress in LTT welded joint.

Apart from the weld toe, the weld root is also a critical position that is susceptible to fracture failure. Hence, it is necessary to evaluate the residual stress development at the weld root. Figure 13 displays the simulated residual stresses at the weld roots of both welded joints after each welding. Obviously, the residual stresses vary after each welding, and the residual Sx stress is greater than the other two stress components in both of the welded joints. For the conventional welded joint, the residual Sx stress decreases from 500 MPa after the first welding to 100 MPa after the sixth welding. Instead, the residual Sx stress in the LTT welded joint has the lowest residual stress of 190 MPa after the first welding, which then increases up to 260 MPa after the sixth welding. On the other hand, both of the residual Sy and Sz stresses in two welded joints decrease after the sixth welding compared to that after the first welding. Additionally, the LTT welded joint has the lower residual Sy and Sz stresses after the first welding, but the residual Sy stress after the sixth welding is relatively higher than that of the conventional welded joint, while the residual Sz stress is similar to that of the conventional welded joint.

Based on the above studies, it can be concluded that the reheat temperature definitely plays a critical role in the residual stress development in a LTT welded joint. In addition, LTT welding materials are more suitable for single-pass welding for tensile residual stress reduction around the weld zone. According to the results of Shiga et al. [36,37], the single-pass LTT elongated-bead method can greatly extend the fatigue life of a corner boxing fillet-welded joint by more than four times without deteriorating the fracture toughness. Hence, there is still a great potential for the engineering application of LTT welding materials.

## 4. Conclusions

Experimental and numerical analyses were performed in order to investigate the residual stress distribution in multi-pass conventional and low transformation temperature (LTT) T-welded joints. It was found that the reheat temperature plays a significant role in the residual stress development of a LTT welded joint. Based on this study, the following conclusions were drawn:

(1) LTT welding materials are more suitable for single-pass welding for the generation of compressive residual stress.
(2) The formerly-formed LTT weld passes with reheat temperatures of up to the austenite finish temperature can produce beneficial compressive residual stress due to martensitic transformation during the cooling process.
(3) The compressive residual stress of the formerly-formed LTT weld bead is converted into tensile stress if the reheat temperature is less than the austenite finish temperature.

## Figures and Tables

**Table 1:** Chemical compositions (%) of the welded plate, the filler wires and their weld metals.

Name	Condition	C	Si	Mn	Ni
**SM490A**		0.200	0.55	1.60	/
**LB-3AD**	Wire	0.034	0.41	3.30	3.30
	As welded	0.067	0.34	2.95	2.65
**LB-47**	Wire	0.080	0.55	0.79	/
	As welded	0.093	0.49	0.64	/

**Table 2:** Parameters of the XRD device used for the residual stress measurement.

**Measuring device**	μ-X360 FULL 2D Portable X-ray Residual Stress Analyzer		
**X-ray tube target**	Cr-Kα	**Diffractive surface**	Fe(α) (211)
**X-ray tube voltage**	20 kV	**X-ray tube electricity**	1.0 mA
**Measuring method**	Single incident angle method	**X-ray incident angle**	45°
**Exposure time**	30 s	**Collimeter spot size**	Standard: Φ 1 mm

## Data Availability

Not applicable.
